# Factors Associated with the Prevalence of Poor Sleep Quality Among Physicians Working in Primary Health Care Centers of the Ministry of Health in Al-Ahsa, Saudi Arabia

**DOI:** 10.7759/cureus.91188

**Published:** 2025-08-28

**Authors:** Zainab M Alamer, Maryam A Alramadhan, Ayat AlNasser, Muslem Albesher

**Affiliations:** 1 Family Medicine, Ministry of Health Holdings, Al Ahsa, SAU; 2 College of Medicine, King Faisal University, Al Ahsa, SAU; 3 Faculty of Medicine, King Faisal University, Al Ahsa, SAU; 4 Family Medicine, Ministry of Health, Hofuf, SAU

**Keywords:** insomnia, physicians, primary health care, psqi, psychological stress, saudi arabia, sleep quality

## Abstract

Background

Sleep is vital for physicians' cognitive performance, emotional regulation, and patient safety. poor sleep quality among physicians may compromise healthcare outcomes and workforce sustainability. However, data on sleep disturbances among primary healthcare (PHC) physicians in Saudi Arabia are limited.

Aim

To estimate the prevalence of poor sleep quality and identify associated factors among physicians working in Ministry of Health PHC centers in Al-Ahsa, Saudi Arabia.

Methods

This cross-sectional study included 196 physicians selected via stratified cluster sampling from PHC centers across Al-Ahsa's four administrative sectors. Data were collected using a structured self-administered questionnaire, including demographic and occupational characteristics, psychological stressors, and the Pittsburgh Sleep Quality Index (PSQI). Descriptive statistics, chi-square tests, analysis of variance (ANOVA), and binary logistic regression were used for analysis, with p<0.05 considered statistically significant.

Results

More than half of the participants (55.1%) had poor sleep quality (PSQI>5). The mean global PSQI score was 6.61±3.56. Poor sleep quality was significantly associated with younger age (particularly 24-29 years, p=0.025), being a general practitioner (p=0.001), emotional stress (p=0.001), bad mood (p=0.001), and loss of interest in usual activities (p=0.001). Logistic regression revealed that age 24-29 years (odds ratio (OR)=7.059; p=0.007) and emotional stress (OR=1.955; p=0.033) were independent predictors of poor sleep quality.

Conclusion

Poor sleep quality is highly prevalent among PHC physicians in Al-Ahsa and is strongly associated with occupational and psychological stressors. These findings call for urgent institutional interventions to promote sleep hygiene, reduce work-related stress, and support physician mental health to ensure safe and effective healthcare delivery.

## Introduction

Poor sleep quality, one of the most prevalent sleep disorders worldwide, is defined by persistent difficulty with sleep initiation, maintenance, or early awakening despite adequate opportunity for sleep, resulting in daytime impairment or distress [1]. As a public health issue, poor sleep quality has gained significant attention due to its widespread prevalence, debilitating consequences, and strong association with both physical and mental health comorbidities. Estimates suggest that approximately 10% to 60% of the general population experiences poor sleep quality at some point, with variations based on demographic, psychosocial, and occupational factors [2]. In Saudi Arabia, it is estimated as the prevalence of poor sleep quality 37% [3]. Among working professionals, particularly those in healthcare, the prevalence of poor sleep quality is disproportionately high due to elevated stress levels, rotating shifts, and emotional fatigue associated with patient care responsibilities [4,5].

Physicians, especially those operating within primary health care (PHC) systems, are uniquely positioned at the frontline of the healthcare delivery chain. In Saudi Arabia, PHC centers form the foundational tier of the healthcare system, and physicians working within them are tasked with extensive responsibilities ranging from preventive care to chronic disease management and acute clinical interventions [6,7]. These clinical and administrative demands often translate into extended working hours, minimal recovery time, and heightened psychological strain, thereby predisposing these healthcare workers to poor sleep quality and poor sleep quality [8].

Poor sleep quality in physicians has far-reaching implications. On a personal level, it is associated with increased risk of anxiety, depression, cardiovascular diseases, and decreased life satisfaction [9]. From a professional standpoint, poor sleep quality compromises attention, decision-making, and memory functions critical to safe medical practice. Several studies have linked poor sleep quality of physicians to medical errors, poor clinical performance, and increased rates of occupational burnout [10]. The combination of cognitive impairment, emotional dysregulation, and fatigue not only undermines physician well-being but also poses a direct threat to patient safety and the overall quality of healthcare services [11].

Despite these recognized risks, research on poor sleep quality among physicians in Saudi Arabia remains limited. A systematic review conducted, which evaluated sleep disorders among physicians, reported a pooled prevalence of poor sleep quality of approximately 37% and noted that nearly half of all physicians experience burnout, often as a result of sleep-related disturbances [12]. Additionally, previous studies from urban centers such as Jeddah and Makkah revealed that between 29.2% and 87.3% of physicians and nurses working in PHC centers suffer from poor sleep quality, highlighting the variability and potential severity of the issue within different regions [4,13]. However, data specific to the Al-Ahsa region - an area with its own unique demographic and healthcare workforce distribution - has not yet been explored, thus leaving a critical gap in the literature.

Multiple factors contribute to the development and perpetuation of poor sleep quality among physicians. These include sociodemographic variables such as age, gender, marital status, and income; occupational variables like job title, shift patterns, and years of experience, and psychological variables including emotional stress, anxiety, and depressive symptoms [14]. Notably, younger physicians often report higher levels of poor sleep quality, potentially due to increased workloads, lack of coping strategies, and career-related uncertainties [15]. Similarly, emotional stressors related to family, finances, or workplace dynamics have been consistently linked with sleep disturbances in healthcare workers [16]. These associations underscore the multifactorial etiology of poor sleep quality and highlight the importance of targeted assessments that incorporate both occupational and psychosocial dimensions [17,18].

The Pittsburgh Sleep Quality Index (PSQI), a validated and widely used self-report instrument, is considered the gold standard for assessing subjective sleep quality and disturbances over a one-month period [19]. It enables the classification of individuals into poor and good sleepers based on a composite score derived from seven sleep-related components. The use of the PSQI in physician populations has revealed alarming trends in sleep quality and has been instrumental in identifying at-risk subgroups [20].

Given the substantial burden of poor sleep quality and its potential consequences for both healthcare providers and their patients, early detection and intervention are critical. Cognitive behavioral therapy for poor sleep quality (CBT-I), stress management programs, mindfulness training, and workplace wellness initiatives have shown promise in improving sleep quality among healthcare workers [21,22]. However, such interventions can only be effectively implemented once the scope and determinants of the problem are well-characterized within specific populations [23-26].

In light of the foregoing, this study aims to assess the prevalence of poor sleep quality among physicians working in PHC centers in the Al-Ahsa region of Saudi Arabia and to identify the key factors associated with its occurrence. By employing a cross-sectional design and utilizing a validated Arabic version of the PSQI alongside a structured sociodemographic and health questionnaire, this research seeks to provide region-specific evidence that may inform future policy-making, occupational health strategies, and clinical interventions. Ultimately, the goal is to enhance the well-being of PHC physicians and promote a safer and more effective healthcare delivery system in Saudi Arabia.

Aim of the study

The primary aim of this study is to assess the prevalence of poor sleep quality and to identify the factors associated with poor sleep quality among physicians working in PHC centers under the Ministry of Health in Al-Ahsa, Saudi Arabia. By employing PSQI and examining a range of demographic, occupational, and psychological variables, this study seeks to generate evidence that can inform targeted interventions to improve physician well-being and sleep hygiene within this critical healthcare workforce.

Research questions

What is the prevalence of poor sleep quality (as measured by PSQI) among physicians working in PHC centers in Al-Ahsa, Saudi Arabia?

Are there significant differences in sleep quality among physicians based on demographic variables such as age, gender, marital status, and nationality?

How do occupational factors (e.g., job title, years of experience, income level, and workplace sector) influence the sleep quality of PHC physicians?

What is the relationship between psychological stressors (e.g., emotional stress, depressive symptoms, bad mood, and loss of interest) and poor sleep quality among physicians?

Which demographic, occupational, and psychological factors independently predict poor sleep quality among PHC physicians in Al-Ahsa?

## Materials and methods

Study design

A descriptive cross-sectional study design was employed to assess the prevalence of insomnia and its associated factors among PHC physicians in Al-Ahsa, Saudi Arabia. This design was chosen because it allows the collection of data at a single point in time, providing a snapshot of the sleep quality of physicians and enabling the identification of potential associations with demographic, psychological, and occupational characteristics. While this approach does not allow for causal inferences, it is well-suited for estimating the prevalence and generating hypotheses for future longitudinal research.

Study setting

The study was conducted in PHC centers affiliated with the Ministry of Health (MOH) in Al-Ahsa. This region, one of the largest in the Kingdom, has a diverse population and a well-structured PHC system. The centers deliver a wide range of preventive, curative, and rehabilitative services and employ physicians with varied specialties, training backgrounds, and years of experience. The setting was deliberately selected because PHC physicians represent the frontline of healthcare delivery and are frequently exposed to occupational stressors that may predispose them to sleep disturbances such as insomnia.

Participants and eligibility criteria

The study population comprised all physicians working in PHC centers in Al-Ahsa during the period of data collection. To ensure methodological clarity and transparency, specific inclusion and exclusion criteria were established. Eligible participants included physicians with permanent appointments in PHC centers who consented to participate. Physicians were excluded if they were on long-term leave, working only temporarily (such as rotating residents or interns), or had a previously diagnosed sleep disorder under treatment. These criteria were applied to minimize the potential sources of bias and ensure that the sample reflected active, practicing PHC physicians.

Sample size and sampling strategy

The minimum required sample size was calculated using the single population proportion formula, assuming a 50% prevalence rate of poor sleep quality (to maximize variance), a 95% confidence level, and a 5% margin of error. The calculation yielded a target sample of 199 physicians. A stratified cluster sampling method was used to enhance representativeness and reduce selection bias. The PHC centers in Al-Ahsa are administratively divided into four sectors - north, south, east, and west. Each sector was considered a stratum, and within each, centers were randomly selected as clusters. Physicians working in these selected centers were invited to participate. This approach ensured coverage of different geographic and service areas while maintaining feasibility in data collection.

Data collection tool

Data were collected using a structured self-administered questionnaire that included two parts. The first part gathered sociodemographic and occupational characteristics, such as age, gender, marital status, years of practice, specialty, and workload variables (e.g., number of patients seen daily, working hours, and shift frequency). The second part assessed sleep quality using PSQI. The PSQI is a widely recognized instrument developed by Buysse et al. (1989) to measure sleep quality over the preceding month. It consists of 19 self-rated items and five questions rated by a bed partner (not included in the scoring). The 19 self-rated items generate seven component scores: subjective sleep quality, sleep latency, sleep duration, habitual sleep efficiency, sleep disturbances, use of sleeping medications, and daytime dysfunction. Each component is scored on a 0-3 scale, yielding a global score ranging from 0 to 21, where higher scores indicate poorer sleep quality. A global score >5 is commonly used to indicate poor sleep quality or insomnia risk.

Although the PSQI has been validated internationally, its adaptation to the Saudi cultural context has been less documented. Therefore, the questionnaire was reviewed by bilingual experts for clarity and relevance, translated into Arabic, and back-translated into English to ensure linguistic equivalence. A pilot test with 20 physicians was conducted to evaluate face validity and internal consistency, resulting in a Cronbach’s alpha of 0.81, which demonstrated acceptable reliability in this context.

This study's questionnaire, which included a translated and qualitatively tested version of PSQI, was first developed and validated by Alomairy (2019). Before it was used, formal permission was obtained. With a reported Cronbach's α of more than 0.78, this version showed satisfactory reliability in Saudi populations [26].

Data collection procedure

Data collection took place between December 4, 2024, and March 18, 2025. Following approval from the regional health authority and ethical clearance. Physicians were approached via email and electronic questionnaire was distributed. To minimize response bias, confidentiality and anonymity were emphasized. The average time to complete the questionnaire was approximately 10-15 minutes. Completed questionnaires were collected in sealed envelopes and securely stored for data entry and analysis.

The study approved by the Local Research Ethics Committee at Prince Saud Bin Jalawi Hospital in Al-Ahsa (IRB No. H-05-HS-135), granted on November 13, 2024.

Statistical analysis

Data were entered and analyzed using the Statistical Package for the Social Sciences (SPSS), version 26 (IBM Corp, Armonk, NY). Descriptive statistics, including frequencies, percentages, means, and standard deviations, were used to summarize demographic characteristics and PSQI component scores. The prevalence of poor sleep quality was calculated using the established PSQI cut-off (>5). Bivariate analyses (chi-square tests and t-tests as appropriate) were performed to examine associations between insomnia and physician characteristics. Logistic regression analysis was then conducted to identify independent predictors of poor sleep quality, adjusting for potential confounders. Statistical significance was set at p<0.05.

Ethical considerations

Ethical approval for this study was obtained from the institutional review board (IRB). Participation was voluntary, and informed consent was obtained from all respondents. Confidentiality was strictly maintained, as no identifying information was collected. Data were stored securely and used solely for research purposes. Participants were assured that declining participation would not affect their employment or professional standing.

## Results

Table 1 presents the demographic characteristics of the 196 physicians who participated in the study. The gender distribution was nearly equal, with men slightly outnumbering women (52% vs. 48%), reflecting a balanced sample that allows for gender-based analysis of sleep outcomes. The age distribution shows a predominance of younger professionals, with 39% aged between 30 and 34 years and 27% in the 24-29 age group. Only a small fraction of participants (7%) were aged 45 or older, suggesting that the PHC physician workforce in Al-Ahsa is largely composed of early- to mid-career professionals, which may have implications for stress and adaptability to night shift work.

**Table 1:** Participant Demographic Characteristics

Characteristic	Group	N (%)
Gender	Male	102 (52)
	Female	94 (48)
Age (years)	24-29	52 (27)
	30-34	76 (39)
	35-39	40 (20)
	40-44	15 (8)
	≥45	13 (7)
Marital status	Married	162 (83)
	Single	28 (14)
	Divorced	6 (3)
Have children	Yes	139 (71)
	No	57 (29)
Salary (SAR)	19,000-24,999	75 (38)
	25,000-29,999	80 (41)
	≥30,000	41 (21)
Smoking	Non-smoker	169 (86)
	Smoker	27 (14)
Chronic diseases	Yes	39 (20)
	No	157 (80)

Marital status data revealed that a substantial majority (83%) were married, while a smaller proportion were single (14%) or divorced (3%). Most physicians (71%) reported having children, a factor that could potentially influence sleep patterns due to familial responsibilities. In terms of income, the majority of respondents (41%) earned between 25,000 and 29,999 Saudi Riyals (SAR) monthly, while 38% earned between 19,000 and 24,999 SAR, and a minority (21%) reported a salary of 30,000 SAR or more. These income brackets are reflective of the typical salary distribution for PHC physicians in Saudi Arabia and may serve as a proxy for job seniority or qualification.

Regarding health-related behaviors, a large majority of participants (86%) were non-smokers, suggesting a generally health-conscious cohort. Similarly, the majority (80%) did not report any chronic health conditions, which reduces the confounding influences of comorbid physical illness on sleep quality.

Table 2 presents the descriptive statistics for PSQI components among the participating physicians. The mean global PSQI score was 6.61±3.56, exceeding the established clinical threshold of 5, which indicates that, on average, the sample experienced poor sleep quality. The wide range (0 to 19) reflects considerable variability in individual sleep experiences. Among the subcomponents, daytime dysfunction (1.27±0.81) and sleep latency severity (1.21±0.97) were among the highest scoring domains, suggesting that difficulty initiating sleep and its impact on daytime functioning were prominent issues among physicians. Actual sleep duration averaged 6.11±1.43 hours, which falls below the recommended minimum of seven hours for adults, further highlighting chronic sleep insufficiency within this population. Notably, sleep latency (the time it took to fall asleep) had a high mean of 28.36 minutes with a striking maximum of 210 minutes, indicating that some respondents experienced extremely prolonged sleep initiation times. The use of sleep medications, though generally low (0.49±0.81), suggests that a proportion of physicians may be resorting to pharmacological aids to manage sleep disturbances. Other aspects, such as sleep disturbance (1.10±0.55) and sleep efficiency (0.93±1.25), showed moderate impairments, suggesting fragmented sleep and reduced time spent actually sleeping while in bed. The low score for perceived sleep quality (0.28±0.69) may suggest that despite quantifiable sleep challenges, participants subjectively rated their sleep more positively - possibly reflecting professional coping mechanisms or underreporting due to role expectations.

**Table 2:** Pittsburgh Sleep Quality Index (PSQI) Outcomes

PSQI Components	Mean ± SD	Min-Max
Sleep latency (min)	28.36±26.71	0-210
Actual sleep hours	6.11±1.43	2-9
Sleep duration	1.33±0.90	0-3
Sleep disturbance	1.10±0.55	0-2
Sleep latency severity	1.21±0.97	0-3
Daytime dysfunction	1.27±0.81	0-3
Sleep efficiency	0.93±1.25	0-3
Overall sleep quality	0.28±0.69	0-3
Medication needed	0.49±0.81	0-3
Global PSQI Score	6.61±3.56	0-19

Table 3 presents the distribution of sleep quality scores, as measured by PSQI, across different age groups of primary health care physicians. The findings reveal a statistically significant variation in sleep quality based on age (p=0.025), with younger physicians reporting notably poorer sleep. Specifically, the youngest cohort aged 24-29 years exhibited the highest mean PSQI score (7.48±3.35), indicating greater sleep disturbances, followed closely by the 35-39 age group (7.40±3.52). Conversely, physicians aged 45 years and older had the lowest mean PSQI score (5.00±2.48), suggesting comparatively better sleep quality. This trend may reflect the combined influence of professional maturity, developed coping mechanisms, and increased life stability among older physicians, who may experience fewer sleep-disrupting stressors. In contrast, early-career physicians may face heightened work-related anxiety, irregular schedules, and career uncertainty, all of which can negatively affect sleep patterns.

**Table 3:** PSQI Score by Age Group PSQI: Pittsburg Sleep Quality Index.

Age group	N	Mean PSQI±SD	p-value
24-29	52	7.48±3.35	0.025
30-34	76	5.87±3.66	-
35-39	40	7.40±3.52	-
40-44	15	6.60±3.79	-
≥45	13	5.00±2.48	-

Table 4 presents the relationship between physicians’ job titles and their PSQI scores, revealing a statistically significant association (p=0.001) between professional role and sleep quality. Among the three groups, general practitioners (GPs) reported the highest mean PSQI score (7.83±3.82), indicating notably poorer sleep quality compared to their counterparts. In contrast, family medicine specialists had the lowest mean score (5.65±2.86), suggesting relatively better sleep quality, while family medicine consultants reported an intermediate score of 6.37±3.72.

These findings may reflect differences in workload distribution, decision-making pressure, and clinical autonomy associated with each professional level. General practitioners, who often serve as the first point of contact in the healthcare system, may experience more stress due to high patient volumes, broader clinical responsibilities, and limited support, which can disrupt sleep patterns. Conversely, family medicine specialists and consultants might benefit from more structured schedules, greater experience, and enhanced coping mechanisms that contribute to better sleep hygiene.

**Table 4:** PSQI Score by Job Title PSQI: Pittsburg Sleep Quality Index.

Job title	N	Mean PSQI ± SD	p-value
Family Medicine Consultant	52	6.37±3.72	0.001
Family Medicine Specialist	75	5.65±2.86	-
General Practitioner (GP)	69	7.83±3.82	-

Table 5 presents a detailed analysis of the association between psychological factors and sleep quality among primary health care physicians, as measured by PSQI. The findings indicate a significant relationship between negative psychological states - namely, bad mood, loss of interest, and emotional stress - and higher PSQI scores, reflecting poorer sleep quality. Physicians who reported experiencing a bad mood during most days of the preceding two weeks had a mean PSQI score of 7.61±3.95, which was significantly higher than those who did not report a bad mood (5.79±2.99, p=0.001). Similarly, those experiencing loss of interest in activities they previously enjoyed had a mean PSQI of 8.11±3.81, compared to 6.04±3.30 among those who did not, also reaching statistical significance (p=0.001). Notably, emotional stress emerged as a critical factor, with stressed individuals scoring 7.30±3.77 versus 5.46±2.86 in their counterparts, again with a highly significant p-value of 0.001. These results underscore the strong association between psychological distress and sleep disturbance among physicians. It is plausible that the emotional burden of caregiving, combined with occupational pressures, may contribute to heightened arousal and impaired sleep regulation.

**Table 5:** Psychological Factors and Sleep Quality

Psychological factor	Group	N	Mean PSQI ± SD	p-value
Bad mood	Yes	88	7.61±3.95	0.001
	No	108	5.79±2.99	
Loss of interest	Yes	54	8.11±3.81	0.001
	No	142	6.04±3.30	
Emotional stress	Yes	122	7.30±3.77	0.001
	No	74	5.46±2.86	

Binary logistic regression identified younger age (24-29 years) and emotional stress as independent predictors of poor sleep quality. Physicians aged 24-29 were over seven times more likely to experience poor sleep compared to those aged 45 or older (OR=7.059, p=0.007). Emotional stress nearly doubled the odds (OR=1.955, p=0.033) (Table 6).

**Table 6:** Logistic Regression Predictors of Poor Sleep Quality

Predictors	OR	95% CI	p-value
Age (24-29 vs ≥45)	7.059	1.70-29.33	0.007
Emotional stress	1.955	1.05-3.64	0.033

Table 7 outlines the frequency of selected additional sleep disturbances reported by primary health care physicians over the past month. Although not part of the core PSQI scoring system, these symptoms provide further insight into the potential contributors to impaired sleep quality within this professional population. Loud snoring was the most commonly reported disturbance, with 6% of physicians experiencing it three or more times per week, an additional 6% experiencing it one to two times per week, and 14% less than once a week; however, a significant portion (44%) reported never experiencing snoring. Leg twitching or jerking during sleep, a possible indicator of periodic limb movement disorder or restless leg syndrome, was noted by 2% of respondents at high frequency (≥three times/week), 6% at moderate frequency, and 9% infrequently, with 54% denying the symptom entirely. Episodes of disorientation or confusion during sleep, which may reflect parasomnias or fragmented sleep architecture, were relatively rare but still present: 0.5% experienced them frequently, 3% occasionally, and 6% sporadically, while 61% denied any such occurrences.

**Table 7:** Frequency of Additional Sleep Disturbances

Sleep Disturbances	≥3 times/week (%)	1-2 times/week (%)	<1 time/week (%)	Never (%)
Loud snoring	6	6	14	44
Leg twitching	2	6	9	54
Disorientation	0.5	3	6	61

## Discussion

The present study aimed to investigate the prevalence and associated factors of poor sleep quality among physicians working in PHC centers under the Ministry of Health in Al-Ahsa, Saudi Arabia. The findings reveal a notably high prevalence of poor sleep quality, affecting 55.1% of the participating physicians. This prevalence aligns with similar studies conducted in the Gulf region, including Jeddah and Makkah, where reported rates of poor sleep quality among healthcare workers ranged from 29% to 87% [27]. The high burden of sleep disturbances among PHC physicians observed in this study underscores a persistent and significant occupational health challenge in this population [28].

Several individual and occupational variables emerged as significant correlates of poor sleep quality. Notably, younger age (24-29 years) and the general practitioner role were significantly associated with higher PSQI scores. These findings are consistent with previous literature, suggesting that younger physicians often experience more work-related stress and less adaptive coping strategies compared to their older counterparts [29]. Early-career professionals may also be more exposed to extended shifts, night duties, and decision fatigue, all of which are established disruptors of circadian rhythm and sleep architecture [30].

The logistic regression analysis identified emotional stress as a strong independent predictor of poor sleep quality. Physicians reporting recent emotional stress were nearly twice as likely to experience poor sleep quality compared to those without such stressors. This finding echoes a large body of research highlighting the bidirectional relationship between psychological stress and sleep disruption [31]. Chronic stress leads to physiological hyperarousal through sustained activation of the hypothalamic-pituitary-adrenal (HPA) axis, which in turn disrupts sleep initiation and maintenance [32]. Moreover, the high prevalence of bad mood and loss of interest among participants - both of which significantly correlated with higher PSQI scores points toward an underlying affective dysregulation that may reflect early signs of burnout or depressive symptomatology [33].

Globally, poor sleep among physicians has been linked to decreased attention span, impaired judgment, slower reaction times, and elevated risk of medical errors [34,35]. Beyond individual consequences, sleep-related impairments in clinical reasoning and patient communication can compromise healthcare delivery quality. A meta-analysis by Philibert confirmed that sleep-deprived residents were significantly more likely to commit diagnostic and procedural errors than their well-rested peers [36].

Interestingly, this study also documented a high frequency of additional sleep disturbances, including snoring and periodic limb movements, albeit in a smaller proportion of participants. These symptoms may suggest comorbid sleep disorders such as obstructive sleep apnea (OSA) or restless legs syndrome (RLS), which are underdiagnosed in the general population and even more so among busy healthcare providers [37]. Future research could benefit from incorporating polysomnographic or actigraphy-based assessments to screen for these conditions more objectively.

The strong association between emotional stress and sleep disturbances found in this study is particularly concerning in light of the growing literature on physician mental health in Saudi Arabia. For instance, a recent study by Almojali et al. revealed that Saudi physicians reported moderate to severe psychological distress during and after the COVID-19 pandemic [38]. This has important implications for long-term workforce sustainability and highlights the urgent need for organizational interventions, including workload management, wellness programs, and mental health support services.

Although this study did not directly assess burnout, the pattern of results - especially regarding mood disturbances and loss of interest - may reflect early features of occupational burnout, a syndrome characterized by emotional exhaustion, depersonalization, and reduced personal accomplishment [39]. Burnout has been widely studied among healthcare providers in the region and is consistently associated with poor sleep quality and impaired sleep [40]. The inclusion of validated burnout measures such as the Maslach Burnout Inventory (MBI) in future studies could enhance the understanding of the interplay between sleep quality and occupational stress.

Limitations

This study has several limitations that must be acknowledged in order to provide an appropriate context for the findings. First, the cross-sectional design limits the ability to establish causality between identified factors and poor sleep quality. While associations were observed, no temporal or directional conclusions can be drawn, and it remains unclear whether occupational and psychological stressors lead to insomnia, or whether poor sleep itself contributes to these stressors.

Second, the study relied on self-reported data, which may be subject to recall bias and social desirability bias. PSQI, although a widely validated tool, captures participants’ subjective perceptions of sleep quality rather than objective physiological measures such as actigraphy or polysomnography. As such, the findings reflect perceived rather than clinically verified sleep disturbances. Additionally, while the PSQI was translated and piloted for clarity, the absence of a fully validated cultural adaptation for the Saudi physician population may have influenced its accuracy in this context.

Third, methodological considerations warrant caution. The response rate, although consistent with physician survey literature, was relatively low, raising the possibility of selection bias. Physicians with poor sleep may have been either more motivated to respond, thereby inflating prevalence estimates, or conversely less likely to participate due to fatigue, leading to underestimation. Furthermore, the modest shortfall in the achieved sample size compared to the calculated minimum is unlikely to have compromised the overall analysis, but it may have reduced statistical power for detecting less common associations.

Fourth, the representativeness of the sample is constrained by the study’s single-region focus. Al-Ahsa’s healthcare system and physician workforce may differ in demographic, occupational, or cultural characteristics from those in other regions of Saudi Arabia. Therefore, the findings cannot be generalized nationally or internationally without caution.

Finally, the statistical analyses were limited. While logistic regression was applied to identify predictors of poor sleep quality, residual confounding remains a possibility given the absence of more detailed modeling of work environment, lifestyle, and psychosocial variables. Future studies using longitudinal designs, objective sleep measures, and broader geographic sampling would strengthen the evidence base and allow for firmer conclusions.

## Conclusions

This study highlights a substantial burden of poor sleep quality among physicians working in primary health care centers in Al-Ahsa, Saudi Arabia, with more than half of the participants experiencing clinically significant insomnia symptoms. Younger age, general practitioner role, and emotional stress were identified as key risk factors contributing to impaired sleep. Furthermore, psychological symptoms such as bad mood and loss of interest were strongly associated with elevated PSQI scores, underscoring the complex interplay between mental health and sleep quality in this population. These findings not only mirror global concerns about physician well-being but also emphasize the urgent need for institutional strategies that address occupational stressors, support psychological resilience, and promote healthy sleep behaviors in frontline healthcare providers. Integrating routine mental health screenings, stress-reduction programs, and workload management policies may offer promising avenues for improving both physician health and patient safety. Future longitudinal and interventional studies are recommended to validate these findings and explore sustainable solutions tailored to the unique context of primary care in Saudi Arabia.
